# Trust and Social Control: Sources of Cooperation, Performance, and Stability in Informal Value Transfer Systems

**DOI:** 10.1007/s10614-020-09994-0

**Published:** 2020-05-20

**Authors:** Claudius Gräbner, Wolfram Elsner, Alex Lascaux

**Affiliations:** 1 Institute for Socioeconomics, University of Duisburg-Essen, Duisburg, Germany; 2 Institute for the Comprehensive Analysis of the Economy (ICAE), Johannes Kepler University Linz, Linz, Austria; 3 ZOE. Institute for Future-Fit Economies, Bonn, Germany; 4 Faculty of Business Studies and Economics, Institute of Economics, University of Bremen, Bremen, Germany; 5 Russian Presidential Academy of National Economy, Moscow, Russia

**Keywords:** Agent-based modelling, Game theory, Hawala, Institutions, Social control, Trust, Value transfer systems, C63, C7, D83, G29, O17

## Abstract

We study the functioning of informal value transfer systems through the example of Hawala. By complementing the institutional theory with computational experiments that use the first agent-based model of IVTS, we examine the roles of generalized trust and social control for the emergence, stability, and efficiency of Hawala. We show that both trust and control are necessary, but not sufficient to guarantee its functioning, and that their relationship is time-dependent. The success of Hawala also depends on population size, interaction density, and forgiveness of the agents. Finally, we provide a theoretically grounded operationalization of generalized trust and social control that is applicable to informal exchange systems in general.

**Electronic supplementary material:**

The online version of this article (10.1007/s10614-020-09994-0) contains supplementary material, which is available to authorized users.

## Introduction

Many financial transactions in emerging economies are arranged on an informal institutional basis. This implies that they cannot be monitored or enforced by the legal authorities and their official regulation is possible only to a limited extent. Examples of these informal financial activities include Rotating Savings and Credit Associations, interlinking agricultural loans, informal value transfer systems (IVTS), and other ‘nonmarket institutions’ (Besley 1995), which are governed by the informal rules that are learned, internalized, and reinforced by the group members. But until now, “very little is known about the mechanisms used by these groups to ensure that members abide by their obligations” (Anderson et al. 2009). Here we seek to explore the governance mechanisms accounting for the emergence, stability, and often surprising success of one of the most significant informal financial institutions, which is involved in informal money transfer around the world and is called *Hawala*.

**Fig. 1:**
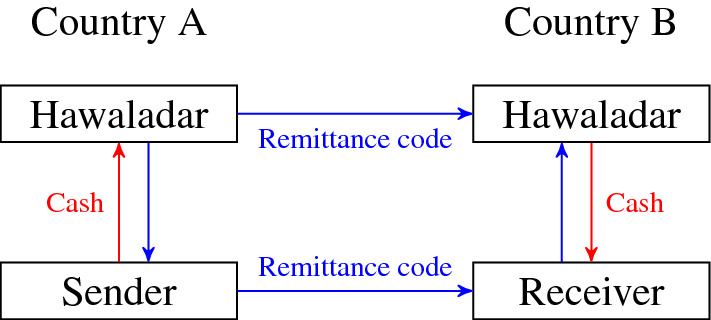
Illustration of the operation of Hawala

People use Hawala to transfer cash from one country to another (see Fig. 1 for an illustration): a sender of money approaches an intermediary called *hawaladar,* handles him a sum of money, and receives a remittance code for transfer to the final recipient. The hawaladar contacts another hawaladar in the target area and informs him about the amount of money to be transferred and a remittance code. The final receiver of money then contacts the second hawaladar, reproduces the remittance code, and receives the money (we explain the functioning of hawala in more detail in Sect. 2). Such a transaction lasts only several hours (or days in case of very remote territories) and hawaladars charge only small commission fees ranging from 2 to 5 percent of the amount transferred. After completing a transfer all traces of the transaction are removed. Estimates of the amount of money transferred through Hawala range from 100 billion dollars (Razavy 2005; Schneider 2010; Schramm and Taube 2003) to as much as 680 billion dollars per annum (Shehu 2004). It is, therefore, considered one of the most important IVTS worldwide (Catrinescu et al. 2009; Rusten Wang 2011).

Given the informality and legal unenforceability of financial claims among Hawala participants, the obscurity and impenetrability of the system’s workings, and abundant opportunities for swindling clients and partner hawaladars out of their money, an important question arises: How does Hawala stabilize the expectations and coordinate the behavior of its participants so as to deter opportunistic defection? The existing literature discusses two major stabilizing mechanisms preventing intermediaries’ opportunistic behavior: generalized trust and social control (Bijlsma-Frankema and Costa 2005; Costa and Bijlsma-Frankema 2007; Das and Teng 2001, 1998; Piccoli and Ives 2003; Sasaki and Uchida 2013).

Until now, however, a number of questions remain unresolved in this literature. In this paper, we try address four of them:

1. How should trust and social control be operationally defined and operationalized?
2. Does trust or social control carry a larger relevance for the functioning of IVTS?
3. Do trust and social control relate to each other as substitutes or complements?
4. How do trust and control relate to boundary conditions, such as group size or interaction density?

Although the most recent studies on Hawala (Fazlur Rahman 2019; Redin et al. 2014; Sharif et al. 2016) add some points to the discussion of trust/control dualism in IVTS, the research questions stated above still remain unanswered. We address these questions by introducing the—at least to our knowledge—first agent-based representation of Hawala. An agent-based approach seems intuitive since it allows us to study Hawala in a *generative* way (see Epstein 1999) and to study the effects of out-of-equilibrium dynamics and shocks—aspects that have so far received little attention in the literature. Since our operationalization of trust and social control is generic, our results generalize beyond the example of Hawala and can be applied to IVTS in general.

Our model specification is mainly built upon *qualitative* evidence, and the outline of the model focuses on the fundamental principles. Thus, our contribution is mainly analytical and we hope that further empirical applications of the model, as well as its refinement through new fieldwork, will improve our understanding of Hawala. But such more complex applications should be left for future research (see also Sect. 7). At this point we introduce the model as a first agent-based representation of hawala that directly portrays the fundamental mechanisms of this value transfer system, that helps to rigorously define and distinguish trust and social control and that enables us to study the behavior of the system out-of-equilibrium. This allows us to contribute a number of theoretical insights to the literature on Hawala, the most important of which we want to preview as follows: First, both trust and social control are *necessary* but *not sufficient* for the successful operation of Hawala. Second, their relation follows a particular *temporal structure*, with trust being especially important for the *emergence* and social control for the *stabilization* of the system. Third, in our dynamic analysis we find that *out*-*of*-*equilibrium* shocks on the trust and control level of the population can have long-lasting effects. Finally, we identify *population size*, *interaction density* and the *forgiveness* of the agents as relevant conditions, which, *together* with trust and social control, are *sufficient* for an efficient operation of the IVTS system. These results, which are discussed in more detail in Sect. 6, complement and extend existing game-theoretical treatments of the subject by directly representing and quantitatively analyzing the important mechanisms regulating IVTS. At the same time, we hope to inspire new empirical research that considers the important parameters identified by our model, and that produces (qualitative and quantitative) data that can then be used to verify and refine the model further.

The remainder of this paper is structured as follows: Sect. 2 describes the key features of Hawala and its functioning. Section 3 elaborates on trust and social control as potential factors of emergence and stabilization of IVTS and informal exchange systems more generally. Section 4 presents our operationalization and the model, which is, to our knowledge, the first agent-based investigation of hawala. Section 5 summarizes the results of our computational experiments, with a broader discussion ensuing in Sect. 6. Section 7 concludes the paper and suggests implications for further research. The dynamics of our model, a more extensive sensitivity analysis as well as additional information about Hawala is provided in the online supplementary material. The program code is openly accessible on GitHub.^1^

## The Functioning of Hawala as an Informal Value Transfer System

Hawala is a venerable, century-old international system of long-range value transfer with its origins traced to ancient China and the Middle East (El Qorchi 2002; Rusten Wang 2011). It continues to operate in a large number of territories and proves its resilience in competition with powerful rivals, such as globally operating official banks, wire transfer companies, and mobile payment services. It is widely used by migrant worker communities that have settled in Europe, North America, and the Gulf region and send remittances to their families in South Asia, Africa, Latin America, and elsewhere (El Qorchi 2002; Rusten Wang 2011). Although often associated with the Muslim culture, nothing in this practice can be specifically related to the Islamic tradition (Parandeh 2009; Razavy 2005). It is open to any customer, regardless of her religious or cultural affiliation, who needs to send or receive money across the borders and wants to get this service done rapidly, inexpensively, and reliably.^2^

Therefore, Hawala maintains a prominent place among IVTS, despite being prohibited in a number of countries (India, Iran, Pakistan) and heavily regulated in others (the UK, the Netherlands, the United Arab Emirates). The fact that Hawala often has to operate in the ‘shadow’ of the law underlines that its functioning is highly dependent on a broad social acceptance of and adherence to its informal rules.

It should be clarified at this point that, although the formal legal system attempts to regulate Hawala operations or even outright forbid them in certain locations, this type of IVTS shows remarkable endurance despite the legal pressures, maintaining its activities mostly in an informal way. While Hawala has been outlawed in some countries, it is de facto tolerated by the authorities. In practice they turn a blind eye on Hawala because of the vast scale of its operations and its importance for financial stability: according to some estimates, remittance flows coming to India through Hawala channels equate to 40 percent of the country’s GDP (Razavy 2005). Due to this ambiguity in the official policy, hawaladars have to operate as a closed community that forms circuits governed by a system of informal norms and conventions. If, alternatively, Hawala is allowed in a certain territory, but is supposed to be heavily scrutinized and regulated by the government agencies, it quickly becomes clear that official monitoring and control can go only so far, given that Hawala transfers can be arranged under assumed names, entries may be non-legible for outsiders, and remittance codes can be highly idiosyncratic. Generally, the system that involves constant accumulation of unaccounted income of dubious origin is to be extremely opaque, so that the authorities should go to great lengths to confirm that a certain amount of money has been transferred from agent A to agent B in the interests of specific third parties and on their behalf, or even that the transaction in question has ever occurred. Therefore, despite all attempts to get Hawala under legal control, this kind of IVTS retains a large degree of informality that determines the processes and relationships within its realm.

Strictly speaking, Hawala does not engage in transferring money between various geographic locations, either physically or electronically. Instead, it arranges a series of swap operations. Any pair of hawaladars has to periodically cancel out their mutual financial obligations. If imbalances persist, outstanding debts between hawaladars can be settled through cash delivery, side payments via conventional banking channels, or even trade arrangements with artificially inflated or depressed prices for imported and exported goods and services (Lambert 2002; Razavy 2005; van de Bunt 2008).

Since Hawala participants cannot go to police, courts etc. with allegations of others’ fraudulent behaviors there exist opportunities, and in fact *incentives*, to cheat and exploit each other. Large sums of money change hands at a word, often with no verifiable records, and hawaladars have to arrange transfers to distant locations prior to, and independently of, their counterparts’ reciprocal moves. Exploitation would give the unilateral defector the maximum possible gain within the transaction, and leave the exploited cooperator with a loss. Assuming that common defection will put them on a par (at the second-lowest payoff), as would common cooperation (with the second-highest payoff possible), this IVTS essentially exhibits the characteristics of a *social dilemma*. In fact, the extant literature has already acknowledged the existence of social dilemma and opportunism problems in the dealings between hawaladars (Schramm and Taube 2003; van de Bunt 2008). The theoretical prediction, therefore, would be that the system gets stuck in the logic of repeated one-shot Nash equilibria, with a relatively inferior performance, and little evolutionary stability among its competing subsystems.

However, in reality Hawala demonstrates considerable endurance and significant competitive advantages over its rivals. So how specifically does Hawala generate and stabilize expectations of trustworthiness and cooperation, and coordinate the behavior of its participants so as to deter opportunistic defection? How exactly does it generate and stabilize informal institutionalized cooperation? Building upon the literature, we envisage two major cooperation-generating, stabilizing, and performance-enhancing mechanisms: emergent *general trust* and systems of *social control*. We will investigate their relative relevance for system performance, including their temporal relationship and crosscheck the two mechanisms against a number of boundary conditions. In doing so, we will focus on the interaction arena of, and relationships among, the hawaladars, thus leaving the examination of client-hawaladar relationships for further research.

## Trust and Social Control as Institutional Drivers of Informal Exchange Systems

Many researchers posit that informal exchange systems, including Hawala, are premised on *trust* (Lambert 2002; Parandeh 2009; van de Bunt 2008). Trust is believed to alleviate the concerns over the delivery of cash, bolster the confidence in the working of the system even in the absence of verifiable records, and allow for long-standing imbalances in the flows of transactions without any request for reclamation and immediate settlement (Lambert 2002; van de Bunt 2008).

Also, *social control* mechanisms have been suggested to stabilize cooperation in IVTS. The instruments of social control that exert pressure on Hawala participants are supposed to be based on dense interconnectedness, the expectations of continuing interactions in the future (Razavy 2005; Schramm and Taube 2003), and the need to protect one’s reputation of integrity (Ballard 2005; Nakhasi 2007).

However, there is no consensus in the literature on IVTS as to what extent trust is relevant compared to social control, or whether they act as substitutes or complements. Our approach in this paper is to a large extent motivated by the conflation of the roles of trust and control as the main coordinating mechanisms in IVTS, which is observed in the extant literature. While some authors maintain that trust serves as a principal enabler of the Hawala transactions (Shanmugam 2004; van de Bunt 2008), others believe that Hawala is stabilized primarily by the use of the social control instruments (Razavy 2005), and yet other researchers of Hawala make no clear-cut distinction between the significance of these two coordinating mechanisms (Ballard 2005; Schramm and Taube 2003). Therefore, in our paper, we attempt to clearly discriminate between the roles of trust and social control in maintaining stable interactions among hawaladars, looking for the answers to the following questions: (1) which instrument, trust or social control, is more important for initiating and sustaining the Hawala transactions, (2) does their relative significance change over time, (3) do trust and/or social control form the necessary and/or sufficient conditions for establishing and developing financial dealings in the Hawala system, (4) do trust and social control work as substitutes or complements to each other?

To solve these important problems, which until now have not been addressed in the literature on IVTS, we will take an evolutionary game-theoretic perspective to develop a theoretical framework and a formal model to rigorously operationalize trust and social control and to clarify their respective roles in IVTS in a computational experiment.

We conceptualize *generalized trust* as the willingness of an agent to cooperate even if she has no information about her counterpart (a ‘stranger’) and knows that it may be a one-shot interaction only, in which the partner has the option (and an incentive) to exploit her. Trust thus implies ‘the willingness of a party to be vulnerable to the actions of another party based on the expectation that the other will perform a particular action important to the trustor, irrespective of the ability to monitor or control that other party’ (Mayer et al. 1995). Such a conception of trust aligns with the approach of the *World Value Survey* (WVS 2015) where ‘general trust’ is measured via questions such as “Do you think you can trust the next person you may incidentally meet?”. Generalized trust is considered to have become learned, habituated, and institutionalized across a critical number of interaction arenas, and thus become independent of a particular interaction context and applied in one-shot interactions even with strangers (Elsner and Schwardt 2014, 2015).

*Social control*, in contrast, is understood as an ability to influence other agents’ behavior through the use of sanctions (Das and Teng 1998). It is well known in the game-theoretic literature that the implementation of effective sanctioning mechanisms is often difficult and impractical since sanctioning is often costly (a second-order dilemma). A form of sanctioning in the case of Hawala, which does not incur significant costs to the sanctioning player, is the exclusion of fraudulent players from further interactions. Such form of sanctioning is particularly effective if the exclusion is not only effected by the exploited agents, but turns into a social sanctioning mechanism applied by several agents. Such a mechanism requires some memory, monitoring, or communication on reputations. A similar operationalization is given by Sasaki and Uchida (2013) in a game-theoretic model in which they provide stability results for equilibria in which fraudulent players get successfully excluded from a cooperative population [related work can also be found in Stanley et al. (1994) and Hauk (2001)]. In Hawala, for instance, defectors may be expelled from the hawaladar community. The threat of ostracism should diminish the potential of opportunistic behavior.

These conceptualizations beg the question of whether the trust-control relationship in informal exchange systems is characterized by complementarity or substitution (Bijlsma-Frankema and Costa 2005; Costa and Bijlsma-Frankema 2007). The *complementarity* perspective (Bachmann 2001; Das and Teng 2001, 1998) implies that trust and social control can mutually reinforce each other, jointly building high expectations in partner cooperation. Agents are supposed to simultaneously rely on socially grounded trusting attitudes as well as reputational and sanctioning mechanisms when forming their expectations. In contrast, the *substitution* perspective (Alvarez et al. 2004; Huemer et al. 2009; Piccoli and Ives 2003) suggests that a higher level of trust comes with a decrease of control, and vice versa. This may even include the possibility of a *mutual crowding*-*out*, when, for instance, social control with its threat of ostracism makes trust redundant (Lascaux 2015), or, on the other hand, increasing general trust would make control superfluous. The different hypotheses are summarized in Table 1.

**Table 1:** Hypotheses about the sources of emergence, stability and efficiency in Hawala, as found in the literature

Hypothesis	References
*The relative importance of trust and social control*	
The emergence, functioning, and efficiency of Hawala is based mainly on generalized trust	Lambert (2002), van de Bunt (2008), Parandeh (2009)
The emergence, functioning, and efficiency of Hawala is mainly due to social control	Ballard (2005), Nakhasi (2007)
*The relationship between trust and social control*	
Trust and social control are complements, i.e. they reinforce each other	Bachmann (2001), Das and Teng (2001, 1998)
Trust and social control are substitutes, i.e. more trust comes with a decrease of control, and vice versa	Alvarez et al. (2004), Huemer et al. (2009), Piccoli and Ives (2003)
The relationship between trust and social control changes over time, with trust being crowded out by social control over time	Lascaux (2015)

To investigate the actual working of the two mechanisms, their interrelation and *relative impacts*, and the potential *time structure* of these impacts, we develop a formal model and run a number of computational experiments with artificial agents interacting under different conditions.

## The Model

The model reflects the functioning of Hawala and of ITVS more generally and provides a rigorous operationalization of generalized trust and social control. This will allow us to test the aforementioned hypotheses on the respective roles of trust and control, and to derive more refined results on their temporal structure and other important factors.

### Model Setup and Parameters

The parameters of the model are summarized in Table 2. We consider a population of *N* agents (hawaladars) that are allocated equally to *M* regions. There are two main types of agents:

**Table 2:** Overview of the parameters of the model. While our initial main interest lies in the effect of trust and social control, we later investigate the effect of the other variables as well

Parameter symbol	Parameter description	Value range (baseline value)
*Main independent variables*		
$\tau$	Trust of cooperators	0 or 1
$\kappa$	Use of control by cooperators	0 or 1
*Control variables*		
*N*	Number of hawaladars	50–1000 (100)
*M*	Number of regions	25
$T$	Number of time steps	750
$I_{max}$	Maximal number of interactions per time step	10–500 (100)
$\gamma_{0}$	Initial share of cooperative hawaladars	50–85% (75)
$\rho$	Resentment period: The number of interactions a cheater will get rejected by those who were informed about her behavior, an inverse of *forgiveness* or *tolerance*	1–100 (10)
$\lambda$	Replication indicator: Percentage of hawaladars who update their strategy	10–20% (15%)

1. *Cooperative agents* will *always* cooperate if they decided to enter an interaction.
2. *Selfish agents* are willing, under certain conditions, to exploit their fellows.

Both types of agents have two behavioral traits: relying on general trust and/or social control. The level of trust and control is specified by the variables $\uptau$ and $\upkappa$ respectively.

For cooperative agents, $\uptau \in \left\{ {0,1} \right\}$ (they may have trust or not) and $\upkappa \in \left\{ {0,1} \right\}$ (they may employ social control or not). Intermediate cases are also of interest, but are not explored here for space constraints (but they can be explored using the openly available source code of the program). Selfish agents always have $\uptau =\upkappa = 1$, as they (logically) always ‘trust’ (hope) that the other one will cooperate in order to exploit her, and as they apply social control since they expect others to potentially cheat on them as well.^3^ The precise functioning of trust and control and our formal operationalizations will be explained below (Sect. 4.2).

### Sequence of Events

**Fig. 2:**
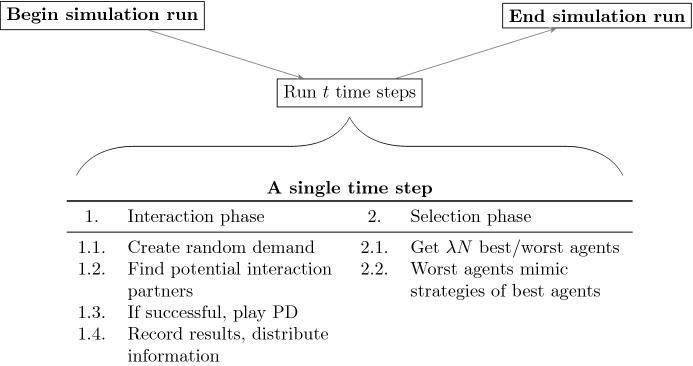
The course of a single simulation. Final results come from Monte Carlo simulations of 50 simulation runs

We analyze our model using numerical simulations, as common in the ABM literature. To this end we simulate the model 50 times and compute summary statistics. Each simulation run consists of 750 time steps, as illustrated in Fig. 2.^4^ Each time step consists of an interaction phase, and a selection phase. Both will be explained in more detail below. This means that the simulation is event-driven in the sense that the single time step corresponds to a certain number of interactions, but not necessarily to particular time period in reality (for more details on event-driven simulations see, e.g., Meyer 2015). Again, this is fairly standard in the literature and not a distinctive feature of our model. At the end of each time step the relevant statistics of the time step get recorded. 750 time steps conclude a simulation run. This general procedure is summarized in Fig. 2.

Each single time step consists of an interaction phase and a selection phase. In the interaction phase, a pre-specified number of interactions takes place. We fix the number of interactions via a parameter since this will allow us to investigate the effect of the ‘interaction density’, i.e. the number of interactions per time step. As it turns out, this interaction density has indeed an important effect on the model outcome. The procedure of each individual interaction is summarized in Table 3.

**Table 3:** Sequence of events for a single interaction

1	Create a random demand, i.e., a money amount to be transferred from one region to another
2	Choose a random hawaladar in the first region
3	Find an interaction partner in the second region
4	The potential partner either accepts or rejects the interaction. If she rejects, the interaction does not take place
5	If the potential partner accepts, agents play a PD as depicted in Fig. 4
6	Record the results of the interaction for both agents

**Fig. 3:**
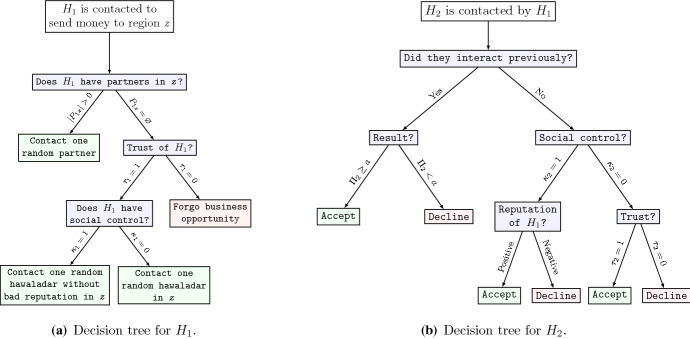
Decision trees for the first hawaladar (the sender, panel **a**), and the second hawaladar (the receiver, panel **b**)

First, a demand for a money transfer service between two regions is created stochastically by choosing two different regions, denoted by *y* and *z*, with uniform probabilities.^5^ Among the agents (hawaladars) located in the first region, one is chosen randomly. This agent will be denoted $H_{1}$. She now needs to find an interaction partner in the second region. Her decision procedure is illustrated in Fig. 3a: She first checks whether she has a *business associate* in the second region (we explain below how agents form business relationships). If she has, she contacts the associate. If she has more than one, she contacts one at random. We denote the set of associates of $H_{i}$ in region *z* as $P_{iz}$.

If $P_{iz} = \emptyset ,$ it depends on her *trust* whether she is willing to contact a stranger for her business: If $\tau_{1} = 1$, she will contact a random agent in *z*; if $\tau_{1} = 0$, she will not engage in this interaction and forgo the business opportunity. If $\kappa_{1} = 1,$ she will not contact any hawaladar that has cheated on her (or one of her business associates) previously.

When a hawaladar in *z,* denoted *H*_2_, is contacted, she can either accept or reject the interaction. The corresponding decision procedure of *H*_2_ is illustrated in Fig. 3(b): If *H*_*2*_ has already interacted with *H*_*1*_ before, and this interaction was positive (*H*_*1*_ did not cheat on *H*_*2*_ and $H_{1} \in P_{2y}$), she will accept the interaction. If the previous interaction was negative (*H*_*1*_ cheated on *H*_*2*_ and thus $H_{1} \notin P_{2y}$), and $\kappa_{2} = 1$, *H*_*2*_ rejects to interact with *H*_*1*_ for $\rho$ periods.

If there has not been any interaction between the two hawaladars before and if $\kappa_{2} = 1$, *H*_*2*_ first checks whether she or one of her business associates was cheated by *H*_*1*_ in the previous $\rho$ periods. If this was the case, she will reject the interaction. In case there is no information about the potential partner, i.e., it is a complete stranger, it depends on the *trust* of the potential partner: If $\tau_{2} = 1$, she will give the interaction a try. If $\tau_{2} = 0$, she will ‘play safe’ and reject the interaction.

At this stage we re-state our operationalization of trust and social control, which we consider to be generic and applicable to any strategic interaction system that involves a population of heterogeneous agents:

*Operational Definition of General Trust* General trust is the willingness to interact with someone one has no information about and who has the potential capability to harm one.

*Operational Definition of Social Control* Social control is the ability and willingness to memorize, monitor, communicate, and ostracize defectors.

These precise operationalizations capture the essence of both trust and social control as discussed previously in Sect. 3.

**Fig. 4:**
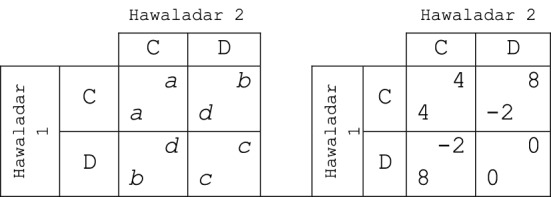
The baseline payoff structure for the underlying prisoners’ dilemma. For the effect of different numerical specifications see Sect. 5.4 and the supplementary appendix

If two agents have agreed to interact, they play a *PD* with the payoff structure as depicted in Fig. 4. As discussed, we formalize the interaction as a PD because it is an ubiquitous incentive structure and decision problem for the agents in any informal exchange system (see Sect. 2 above).

The particular payoff structure, however, deserves explanation. We chose the values to resemble the real situation of hawaladars. The payoff of *mutual defection* should be zero, i.e., benefits and losses would be mutually balanced between two interacting agents.^6^ So the only parameters we have for setting the *fierceness of the PD* are the remaining payoffs *b*, *a*, *d.* To see how severe the dilemma structure can be to still allow for cooperation see Sect. 5.4 and the sensitivity analysis in the supplementary material.

After an interaction, the agents are awarded their payoffs (denoted by = $\varPi_{i}$), record the relevant information about the interaction, and adapt their settings and behaviors accordingly: If $\varPi_{i} > 0$ agent *j* becomes an associate of *i* and vice versa. Otherwise (if $\varPi_{i} \le 0$), *i* will remember *j* as a defector and will reject her the next $\rho$ times (see Sect. 5.3 on the degree of forgiveness). Furthermore, if $\kappa_{i} > 0$, *i* also informs all of her associates about *j’*s defection. They all will then reject *j* (for $\rho$ interactions) as if they had been exploited by her themselves.

When all interactions have taken place, agents change their strategies according to social learning: agents change their strategy if they perform particularly badly, i.e., belong to the $\lambda N$ most unsuccessful agents in terms of accumulated payoff. The probabilities for their new strategies to be chosen shall be equal to the distribution of strategies of the $\lambda N$ most successful agents.

We tested for several criteria to determine the relative success of agents, for example, ranking them according to their *total accumulated payoff*, the *payoff in the previous time step*, or the *average payoff* across a number of time steps. The effect of the particular measure chosen is marginal compared to other mechanisms driving the outcome. Also, there are no qualitative changes dependent on the particular value of $\lambda$. We thus chose $\lambda = 15\%$ as the default.

## Results

### The Respective Impacts of Trust and Control

To clarify the respective roles of trust and social control and scrutinize the first pair of hypotheses in Table 1 we compare four baseline constellations:

1. Cooperative agents have $\tau = \kappa = 0$, so neither do they have trust nor do they use social control.
2. Cooperative agents have $\kappa = 0$, but $\tau = 1$, so they have trust but do not use social control.
3. Cooperative agents have $\kappa = 1$ but $\tau = 0$, so they use social control but do not have trust.
4. Cooperative agents have $\tau = \kappa = 1$, so they have trust and use social control.

In order to judge the effects on the functionality of the system, the following four state variables of the system are of particular interest:

(i) share of successful interactions,
(ii) type of interactions that have taken place (i.e., mutual defection, exploitation, or cooperation),
(iii) final share of cooperators,
(iv) efficiency of the system; efficiency is defined as the total realized payoff divided by the maximum payoff possible, i.e. the total wealth that would result if all potential interactions would have been carried out as mutual cooperations.

**Fig. 5:**
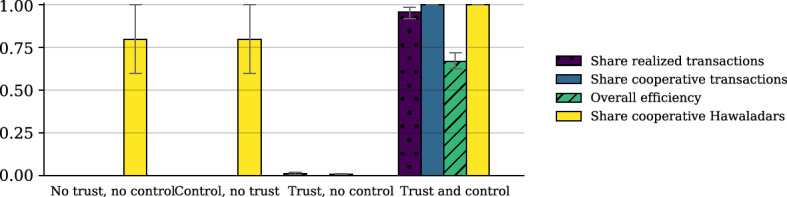
Results for the roles of trust and control—illustration. The graph shows the means and 10th and 90th percentiles of 50 simulation runs after 750 time steps with the baseline specification as shown in Table 2

All results will be displayed by the mean and the 10/90-percentiles. For the baseline analysis, we analyze 50 simulation runs, which do not show much inter-run variation. Figure 5 summarizes the results after 750 time steps. The dynamics of adjustment are illustrated in more detail in the *supplementary material*.

The results indicate that *both trust and control are necessary* for the system to function properly:

1. *Without both trust and control* we observe a complete breakdown of the system: no interactions take place and almost no payoffs are realized. Consequently, the further selection of strategies is completely random since no agent accumulates payoffs and can be considered more successful than others.
2. A similar result occurs if cooperators use *social control, but do not have any trust*. In this case, the system does not take off either: Since no cooperator has trust, they do not form any relationship among cooperators, and only the selfish agents actually operate in the beginning. But using social control, the activity of the selfish agents gets suppressed quickly so that after a short period no interactions take place at all.
3. If cooperators have *trust but no control*, they interact naively also with known defectors, get exploited and extinct. The few realized interactions are mutual defections among selfish agents, and the system remains highly dysfunctional.
4. If cooperators *use social control and have trust*, the system approaches a state of considerable efficiency: selfish agents are crowded out of the system, almost all interactions take place, and on average almost 75% of the potential payoff can be realized.

In principle, the model also allows to study intermediate cases, such as situations in which $\alpha$% of the population has trust and $\left( {1 - \alpha } \right)\%$ does not. Alternatively, one could study a situation in which agents cooperate with a given probability, the latter potentially being dependent on the past success of the agents. But we decided to leave these experiments to future research (or the reader) and instead want to explain how we can study other parameters of interest in our model. To this end, we now turn to the temporal structure of trust and control.

### The Temporal Structure of Trust and Control

We test the second set of hypotheses outlined in Table 1, which refer to the dynamic relationship of trust and control. Lascaux (2015), for instance, suggested that trust is only important in the beginning, but crowded out by social control over time. To test this, we ‘shock’ the system by exogenously setting the trust or control values for cooperators to zero after a particular number of time steps. At this point, it is important to keep in mind that time in our simulation is *event*-*based time* rather than *real time*: one time step consists of a fixed number of interactions, no matter how long they take in real time. This means that our results relate to the maturity of the system in the sense of the number of interactions that have taken place so far.

**Fig. 6:**
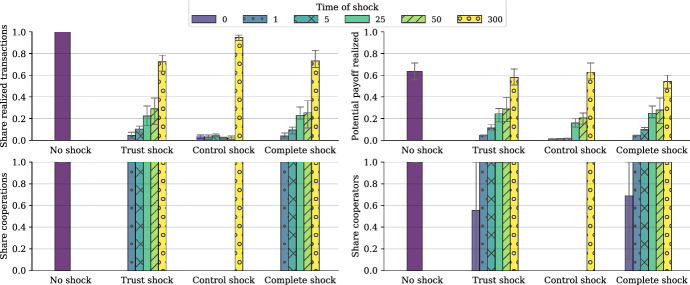
The effects of trust and control shocks at different time steps. The figure shows the means of 10 simulation runs. Whiskers again indicate the 10th and 90th percentiles and parameter specifications are given in the baseline column in Table 2

Our results on the temporal structure of Hawala are illustrated in Fig. 6. The single bar on the left of every panel refers to the case where no shock affects the system. Bars indicating the results for a shock at time step zero are equivalent to runs where no trust or control operate at all. These cases serve as ideal benchmarks to facilitate the interpretation of the other outcomes.

The first observation confirms the importance of the timing of shocks: we see that shocks after 300 time steps have little or no effect since the system already settled into a stable equilibrium. However, earlier shocks that reach the system out of its equilibrium can have profound and self-reinforcing effects.

**Fig. 7:**
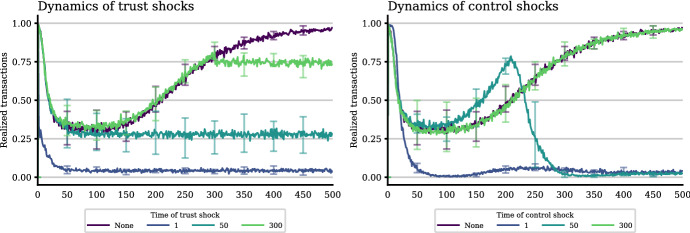
Adjustment dynamics for the share of realized transactions for different timings of shocks. The results are the averages of 20 simulation runs. Whiskers again indicate 10th and 90th percentiles. The parameter specification is the baseline specification in Table 2

At least for the trust and complete (trust plus control) shocks, it holds that the earlier the shock, the more profound and persistent its impacts. The reason why earlier trust shocks have more profound effects is straightforward: in the beginning agents do not know each other. They can form new relationships only if they trust strangers. Once trust gets eradicated from the system, no additional relationships can be formed and successful transactions only pass through the (few) relationships already formed. Thus, even if after a trust and complete shock all agents end up being cooperative (see lower right panel) and all realized interactions are cooperations (lower left panel), the share of realized transactions gets significantly reduced (upper left panel) and the efficiency of the system goes down accordingly and reaches obviously unsustainable values (upper right panel). The fact that a trust shock after 300 time steps still reduces efficiency of the system, indicates that at this point not all agents have formed partnerships with each other (see Fig. 7, left panel, for the dynamics of this process). Consequently, it makes a difference for the system whether there is no trust at all—in which case the system breaks down—or trust gets eradicated after some time. In the latter case the system works inefficiently (and, presumably, not sustainably), but does not break down completely. These results align with the argument in Lascaux (2015), but the conclusion is somewhat more specific as we do not observe a complete crowding-out of trust. Trust is, and remains, the directional guide for the process, on which interactions become feasible.

*Social control*, then, may stabilize, qualify, but also restrict this process: Every *control shock* before the complete eradication of defective agents can cause the system to break down completely, because in this case the short-term gains of the defectors—who are now more easily able to exploit cooperators—are larger than those of cooperators, and defectors take over the population (see Fig. 6, bottom right panel). However, once there are no defectors in the system any more, also social control becomes obsolete and there is almost no difference to the case of no shock at all. See Fig. 7 (right panel) for the dynamics, in particular the difference to a trust shock after 300 time steps.

The similarity of the results for the trust and complete shocks is surprising. It suggests that somehow trust ‘trumps’ social control: The eradication of trust after some time can even serve as a (imperfect) substitute for social control. Once trust is eradicated, there is virtually no situation in which cooperators could be exploited: If a cooperator is chosen for a transaction, she will again contact her associates—who are unlikely to become defectors—and will cooperate with them. If she does not have an associate for this interaction she will—because of her lack of trust—forgo the business. The same is true if she is approached by an agent she has no direct information about. The need for social control in such a setting is greatly diminished and the mere *absence of trust serves as a substitute for control*—at least in the protection of cooperators that have already established a number of working relationships. However, the resulting system is still inferior (as cemented at its status quo of relations existing prior to the trust shock) compared to the situation in which both trust and control operate.

In all, trust and social control exhibit a *clear temporal pattern*, which provides us with insights into the mechanisms themselves and their interrelations. Basically, trust and control display some particular complementarity: Existing trust establishes the need for control, later trust may be somewhat dispensable; but only if both are operating simultaneously, the system can realize its potential.

### The Importance of Favorable Boundary Conditions

So far we have shown that trust and control are *necessary* for the success of informal exchange systems, and that they are related in a certain temporal structure. We will now see, however, that they are *not sufficient* to ensure a sustainable and successful (let alone an efficient) functioning of the system. There are other factors that are essential for its success as well. Other than for trust and control, however, less favorable conditions of one factor can, to some extent, be compensated by more favorable conditions of another: in contrast to trust and control the factors discussed here are to some extent substitutable. We discuss three factors: the number of agents, interaction density, and the ‘forgiveness’ of agents.

#### Population Size: More Agents—More Trouble

**Fig. 8:**
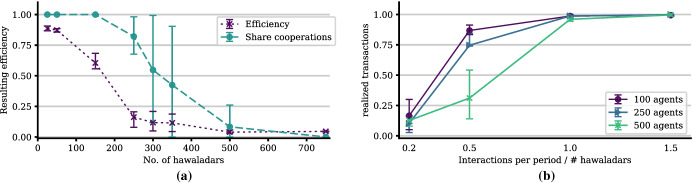
Number of agents that the system can successfully accommodate (**a**). The higher the interaction density, the more agents can be accommodated by the system (**b**). The other parameters are as given in Table 2

As illustrated in Fig. 8a, more agents, *ceteris paribus*, reduce the efficiency of the system until it collapses. Too many agents prevent the mechanism of social control to function properly as cooperators cannot gather enough information on potential defectors in comparison to the increasing population size. The requested knowledge about a new interaction partner’s past behavior simply cannot be discovered and disseminated fast enough.

This result comes with a natural interpretation in the context of Hawala: The system can—*ceteris paribus*—accommodate only a limited number of agents successfully through its self-organization mechanisms, due to the underlying *cognitive conditions* of *expectations building*, *memorizing*, *monitoring* and *information diffusion*, as increasing degrees of *anonymity* and *uncertainty* accompany increasing population sizes. Note that we deal with the *relevant population* size, the population or group within a delimitable interaction arena. Obviously, this relates to the factor of *arena* or *group size* (see Elsner and Schwardt 2014, 2015).

These results, however, beg the question of what determines the number of hawaladars the system can actually accommodate successfully. It turns out that this missing factor is the relative interaction frequency.

#### Higher Interaction Density Favors Cooperation

As we can infer from Fig. 8b, the more *interactions per period* take place—*ceteris paribus*—the more agents the system is able to accommodate while maintaining a high performance. The intuition underlying this result is similar to the previous one: lower interaction density reduces the ability of the agents to gather information, ultimately used for *social control*. These results align well with previous game-theoretic and evolutionary-institutional modeling of the cognitive, communication, and reputation-related mechanisms required for the emergence of cooperation (Elsner and Schwardt 2014, 2015), and were corroborated in anthropological, psychological, neuro- and brain-sciences (see, e.g. Henrich et al. 2004 and Gintis 2007).

#### Forgiveness Makes the System More Efficient

After an agent gets exploited, she and her associates will reject the next $\rho$ interactions with the exploiter. The number of rejected interactions, until this exploiter is given a new chance, the *retaliation period*, is the inverse of forgiveness, the *propensity to take up cooperation again* by a cooperative agent. This could also be considered the degree of *tolerance* of former defections.

**Fig. 9:**
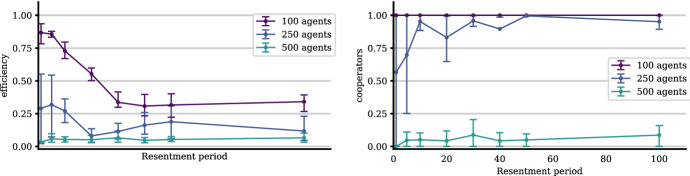
The role of forgiveness. Note that forgiveness does not have an impact on the resulting relative level of cooperation but only on the efficiency of the system because fewer interactions are realized. The other parameters are as given in Table 2

Retaliation/forgiveness has an important effect on the efficiency of the system: If agents do not forgive former defectors, i.e., remember them and refrain from interaction with them for too many periods, the system cannot realize its full potential: former exploiters who have become cooperators do not get reintegrated. Consequently, potential gains from cooperative interactions are not realized (see left panel of Fig. 9). The reason is not that fewer agents are cooperative, but that fewer interactions are realized (see right panel of Fig. 9).

### Sensitivity Analysis

A potential drawback of agent-based modeling is some lack of transparency (see Gräbner 2018), which may quickly occur in simulating complex systems. In our case, however, we can explore the effects of all free parameters on outcomes, as we kept the model relatively simple and thus maintained a sufficient level of transparency. We summarize the results in Table 4. Even more detailed sensitivity analyses are provided in the supplementary material.

**Table 4:** Summary of the effects of parameter changes

Parameter	Value, value range	Effect on outcome	Details can be found in…
Number of agents	50–1000	More hawaladars make it—*ceteris paribus*—generally more difficult for cooperation to emerge. A larger interaction density can compensate this effect	Section 5.3.1
Initial share of cooperators	25–85%	Cooperation emerges for values above 50%. *Ceteris paribus,* the higher the share, the quicker the equilibrium is reached	Supplementary material
Interactions per period (interaction density)	10–500	*Ceteris paribus,* more interactions per period favor cooperators. Too low values prevent cooperation to emerge	Section 5.3.2
Rejection period (inverse of forgiveness)	1–100	Affects mainly the efficiency of the system: too large values reduce efficiency	Section 5.3.3
Percentage of agents who update their strategy after a time step	10–20%	Only affects the speed of adjustment towards a particular equilibrium	Supplementary material
Fierceness of the dilemma	$$ \left| {{\raise0.7ex\hbox{$a$} \!\mathord{\left/ {\vphantom {a d}}\right.\kern-0pt} \!\lower0.7ex\hbox{$d$}}} \right| \in \left( {0.5, 5.5} \right) $$	For reasons explained, we keep c = 0. Then, for d = − 2 or d = − 4, we test for different relations of |a/d|, keeping b = 2a. Results remain stable for most specifications, but adjustment periods take longer if dilemma is fiercer (see the supplementary material)	Supplementary material

The central result that both trust and control are *necessary* for Hawala to function holds under all reasonable parameter specifications, and is subject only to the dynamic (temporal) results outlined above (Sect. 5.2). However, certain parameter constellations make it considerably more difficult for cooperation to emerge, so that the emergence of a functioning system—even if trust and control exist—is not guaranteed. We summarize the relevant conditions in Table 5.

**Table 5:** Summary of the necessary and sufficient conditions for Hawala to function and of the impact of the important parameters

*Necessary conditions:* need to be present for the system to function at all	*General trust:* willingness of cooperative hawaladars to interact with strangers
	*Social control:* willingness and ability of cooperative hawaladars to monitor and exclude fraudulent hawaladars
*Other important conditions:* must jointly provide a sufficiently friendly environment for the system to function	*Size of population:* absolute number of hawaladars may not be too large
	*Interaction density:* number of interactions per period is sufficiently large
	*Forgiveness:* period in which former defectors are excluded is not too long

As the code for the model is freely available online, the validity of the results may be tested and the model can be easily extended to address further questions. We illustrate how this can be done in the next section.

### Extensions

Our model is built in a *modular* way. This means that it is relatively straightforward to extend it and to study the effect of factors not considered in the original—admittedly rather simple—model. We illustrate the usefulness of such modularity by introducing two extensions: population growth and decision mistakes.

#### Population Growth

**Fig. 10:**
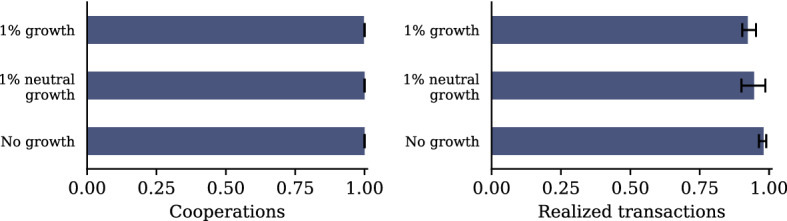
Simple population growth has no significant effect on the model outcome

One might expect the model results to change significantly once population growth is allowed for. To test this hypothesis, we extend the model—leaving everything else unchanged—by introducing two forms of population growth: ‘Neutral’ growth adds new agents with a random strategy, matching the initial distribution of strategies. ‘Normal’ growth adds new agents such that there is a 50% chance for them of being cooperators or defectors. As can be seen from Fig. 10, such population growth does not change the functioning of the system significantly, only ‘normal’ growth has some very small disturbing influence on the strategy distribution because the newly entering hawaladars have a different strategy distribution than the ones already in the population.

However, more complex processes of population *turnover* with many entries and exits may have an impact on the system’s efficiency, e.g., when less successful agents do not (or cannot) change their strategies quickly enough, but leave the population, thus corrupting the information diffusion system.

#### Random Mistakes (‘Trembling Hands’)

**Fig. 11:**
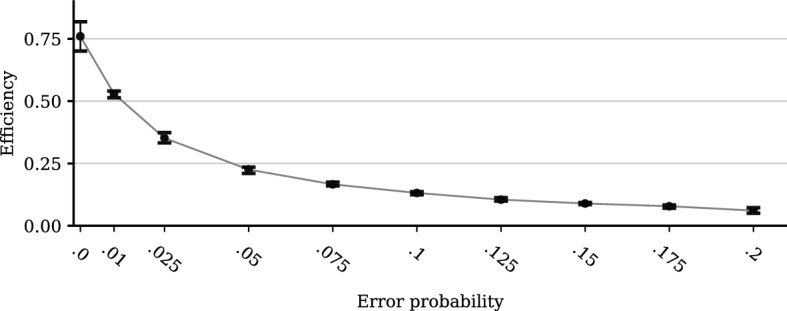
The effect of stochastic mistakes. Error probability denotes the probability that a cooperative hawaladar cheats by accident, and a selfish hawaladar cooperates by accident

Another insightful way to extend the model is to allow agents to make mistakes. Here we consider purely stochastic mistakes: With a given probability, cooperators defect upon their interaction partners, or selfish agents cooperate. We might expect that such mistakes have a severe impact on the system since they erode partner selection and, with this, the social control mechanisms. If a cooperative agent defects by accident, she will not be able to interact with her interaction partner and all of the partner’s business associates. The results shown in Fig. 11 confirm this expectation: Even small chances of mistakes significantly reduce the efficiency of the system.

## Discussion

With our computational experiments we contribute to a deeper understanding of the working principles of informal economic exchange systems and of the pathways through which two important institutional self-organization mechanisms, general trust and social control, exert an impact on the systems’ functioning. Given opportunities (and often incentives) for agents’ fraudulent behavior in a complex, apparently opaque and unenforceable system such as Hawala, the question of how participants coordinate and stabilize their expectations and behaviors and achieve relatively high performance results is of prime interest. The literature has considered the role of general trust and social control in informal economic interactions, but so far, it has been unclear,

1. how the two should be operationally defined and operationalized formally,
2. which, if any of the two, carries a larger relevance for the functioning of IVTS,
3. whether (and when) they relate to each other as substitutes or complements, and
4. how they interact with a number of other boundary conditions.

Our model, which, to our knowledge, is the first agent-based representation of IVTS, particularly Hawala, makes a case for an essentially equal significance of trust and control: In our computational experiments, it was impossible for the system to emerge if either one was completely missing. This supports a basic *complementarity* argument.

In the context of elaborating their *temporal interaction*, our model was helpful in elucidating the mechanisms underlying this relationship: Put simply, general trust is the channel for the emergence of a functioning system, while social control is needed to protect the system in its further evolution against the threats of opportunism.

We also derived specific results concerning the trust/control dynamic relationship. Both are essential at the beginning of the interactions. After a few relationships have been formed, trust could basically be eradicated from the system without a complete breakdown, but the efficiency of the system would remain considerably low. Trust would become redundant, while performance remained high, only if all agents would know each other from previous interactions. This, however, is unlikely to arise in practice since real-life systems are characterized by an ongoing turnover of agents, incompleteness of information, and other imperfections (of memorizing, monitoring, reputation building, communication, and information diffusion, or random mistakes). Similarly, social control is important as long as the population of agents includes fraudulent players. We found no evidence of a complete crowding-out of either trust or control, with one exception: The absence of trust could serve as an imperfect substitute for social control at a later phase of the development. But this would come at the cost of decreasing efficiency.

We also studied the sufficiency conditions for the system to work properly. While trust and social control are necessary, some other boundary conditions must be sufficiently favorable to allow for reasonable performance and stability. Conditions we have explored in this respect are population size, interaction density, and forgiveness. Here (and in contrast to trust and control), less favorable values for one condition can—to some extent—be offset by more favorable values for other conditions. We found our results to be robust and illustrated the possibility to extend the model by studying, as examples, the effects of population growth (which are negligible) and the possibility that agents make mistakes (which is significant).

While developed for the case of Hawala, our model provides some more general insights. For instance, we explored an operational definition of trust for analytical and computational models. Our definition not only captures the essential elements of previous definitions of trust and social control, it also aligns well with empirical conceptualizations such as in the *World Value Survey.* When it comes to social control, our definition is also easy to operationalize in both analytical and computational models.

At the same time, it is important to note that the experiments in this paper are only a first step of using the model. In many ways we kept the assumptions rather simple, in order to introduce the model and its basic mechanisms more clearly. It is clear that Hawala in the real world is a very complex system. And while comparing our model with qualitative descriptions of Hawala suggests that it captures Hawala’s key mechanisms, the model needs to be refined and verified further before real-world implications can be derived from it. Nevertheless, our results tend to suggest some interesting policy implications: policies aiming at the stabilization (and high performance) of cooperation, should focus on creating boundary conditions that support the emergence of trust and social control, and therefore self-organization and self-governance without formal/legal intervention. Such a policy leaves the space for adaptation and evolution taking place in the system of informal interactions among private agents. But while this suggests that computational analyses of informal economic exchange systems do have important real-world applications and policy relevance, certainly more empirical research is required before our model should inform such policies in practice.

## Conclusion and Further Research

In this paper we contributed analytically to the understanding of IVTS and the functioning of trust and social control in such systems. Our model has been rather simple as it is necessarily only a first step to study Hawala using computational experiments. Yet, the model is openly available, easy to extend and, thus, invites several lines of further research, some of which may be pursued with the computational model itself, while others may build upon the hypotheses that we have derived from it.

An immediate avenue for future research that makes use of the model is to investigate the ‘intermediate’ cases of trust and control, i.e. those cases in which the trust and control levels of cooperative hawaladars lie between zero and one. Another easy to implement but interesting extension would be to change an agent’s trust towards strangers as a reaction to being exploited.

Another opportunity is to introduce more heterogeneity to the agents and regions: one could study the effects of asymmetric transactions flows, i.e. situations where flows from one group of regions to the other are more likely than the other way around, or how a heterogeneity of, e.g. risk aversion of the agents affects their relative success and the overall dynamics of the system.

A further avenue would be to link the model directly with data from field studies: after having obtained quantitative data about the framework factors discussed here (such as population size, interaction density, number of hawaladars in specific regions, etc.), one could calibrate the model to this data and thereby verify its results further. Moreover, fieldwork likely suggests other parameters to be included in the model, such as wealth of the agents involved, their education or other cultural background variables. In the best case, this leads to a constant feedback between the fieldworker and the user of the model, such that through their joint effort our understanding of IVTS can be further enhanced.

Finally, we did not include the clients’ level in IVTS. But local value transfer agents (hawaladars) and clients do have certain interaction structures as well, and even sending and receiving clients may display non-trivial interrelations between themselves. The exploration at this level would, however, require some empirical data, field studies and/or analyzing the features of specific IVTS. Collecting and processing related data will, in general, be an important task for future research of informal exchange systems, and the direct interaction between fieldworkers and modelers. The modeler implements the data and information from fieldwork into a computational model and may put more concrete questions to the fieldworkers, who then can obtain new and more specific data. This appears to be an effective way to make progress on this front.

Because our model is freely available, we may invite researchers to scrutinize and extend the software to address these issues. But studying the subject with a different methodological approach and relating the results to each other seems to be equally beneficial. In any case, our study has shown that informal economic exchange systems depend crucially on social factors. By explicating the mechanisms through which trust and control perform the functions of coordinating and stabilizing the expectations of IVTS participants, we hope to have enlightened some of the mechanisms underlying this dependency.

## Electronic supplementary material

Below is the link to the electronic supplementary material.

Supplementary material 1 (PDF 872 kb)

## Data Availability

All code used to create the results of the model is freely available via Github: https://github.com/graebnerc/trust-control-hawala.

## Footnotes

1 See https://github.com/graebnerc/trust-control-hawala.

2 A more detailed discussion of the competitiveness of Hawala is provided in the online supplementary material.

3 At first sight, it may seem counter-intuitive to argue that selfish agents have trust and use social control, as both are mainly considered mechanisms to foster general cooperation. But trust as defined here captures the willingness to interact with strangers. Since selfish agents are willing to exploit others they have an interest to interact with strangers, as these are easier to exploit. Considering social control, since selfish agents are willing to exploit others, they would expect others to do this as well and would therefore be more skeptical of the behavior of others, thus employing some control.

4 We chose 750 time steps because after this period we can be assured that the model has reached its equilibrium. We speak of an equilibrium once we cannot observe any relevant further change in the state variables of interest. This usually happens after 450 time steps. The concrete dynamics are shown in the online supplementary material.

5 We choose the regions from a uniform distribution for two main reasons, one of which is pragmatic and one substantial: first, on a pragmatic level, we want to keep our results as generic as possible and it is clear that any deviation from uniform probabilities requires careful justification. Such a justification should refer to the particular application of the model. Since we here use the model as a general representation of Hawala, it seems more plausible to focus on the base case of uniform distributions. Second, there are also substantial reasons justifying the uniform distribution: Hawala operations are spread across the vast territory encompassing Europe, Middle East, South and South-East Asia, Africa and both Americas and are supported by a broad network of hawaladars scattered across the globe. Migrants from South and South-East Asia, Africa and South America work in Europe, North America and Middle East and need to remit their savings back to their families. Informal value transfers, therefore, need to be supported on both sides of the chain, at the points of sending and receiving money, for hawaladars to be able to serve both migrants and their kin. Under these circumstances it is difficult to justify an alternative to the uniform distribution (see Shanmugam 2004 or Razavy 2005 for more details on the distribution of Hawala services).

6 Even if *c* would be negative, the qualitative results of simulations of the model would not change. Only if $c > 0$, the dynamics would change; but it does not make sense to think of mutual defections being more favorable than no interaction at all.
