# Supplementary material for: Trust and Social Control: Sources of Cooperation, Performance, and Stability in Informal Value Transfer Systems
Source: Comput Econ. 2020 May 20;58(4):1077–102. doi: 10.1007/s10614-020-09994-0 (PMC8589791; doi:10.1007/s10614-020-09994-0)
Supplement: Supplementary file 1 — Supplementary material 1 (PDF 872 kb) [file 10614_2020_9994_MOESM1_ESM.pdf]

# Trust and Social Control

## Sources of cooperation, performance, and stability in informal value transfer systems

### *Supplementary material*


Claudius Gräbner[a,b,c*] Wolfram Elsner[d] Alex Lascaux[e]

[a] *Institute for Socioeconomics, University of Duisburg-Essen, Germany*
[b] *Institute for the Comprehensive Analysis of the Economy (ICAE), Johannes Kepler University Linz, Austria*
[c] *ZOE. Institute for Future-Fit Economies, Bonn, Germany*
[d] *Institute of Economics, University of Bremen, Germany*
[e] *Russian Presidential Academy of National Economy, Moscow, Russia*
[*] *Corresponding author: claudius@claudius-graebner.com*



**Abstract**

Here we provide more detailed information on the dynamics of our model (S1) and present the results of a more extensive sensitivity analysis (S2). We also provide a summary of reasons for why people might prefer hawala over formally established alternatives such as formal banks (S3).

## *S1 The dynamics of hawala*

Figures A1 and A2 illustrates the dynamics of the model for the four baseline cases discussed in section 5.1. As one can see, the model does not show much inter-run variation. The only exception is the share of cooperators in the cases where neither trust nor social control are present. The reason for this has been discussed in the paper: Since there are almost no successful interactions, no type of agent persistently outperforms the other. When agents change their strategy (i.e. cooperative hawaladars become selfish and vice versa), no clear pattern emerges.

*Figure A1*

The adjustment dynamics for the first two baseline cases.

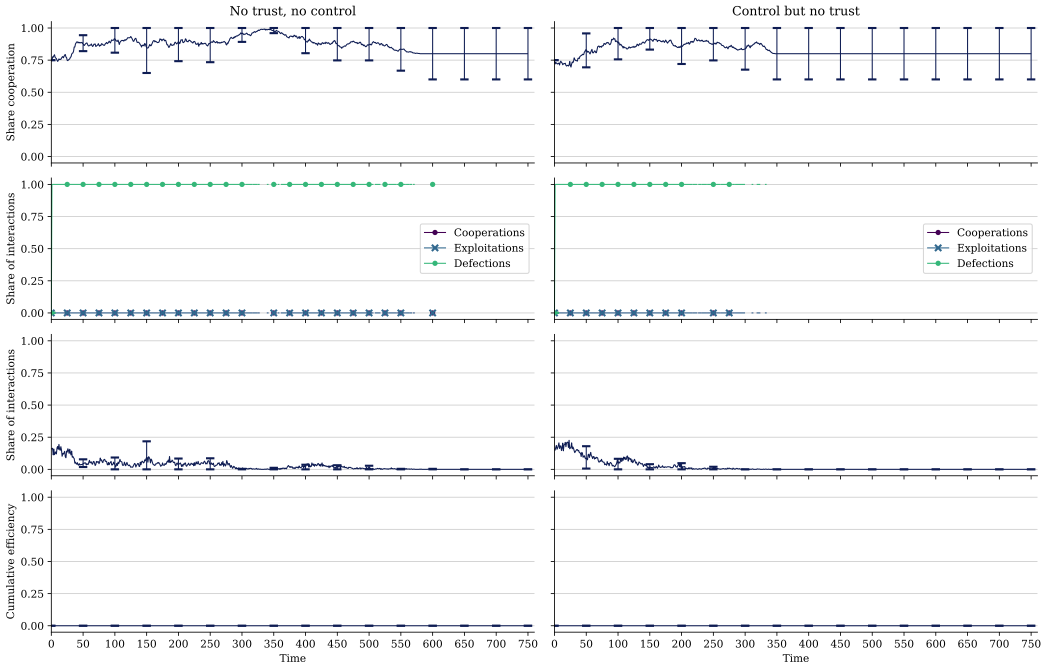

*Figure A2*

The adjustment dynamics for the third and fourth baseline cases.

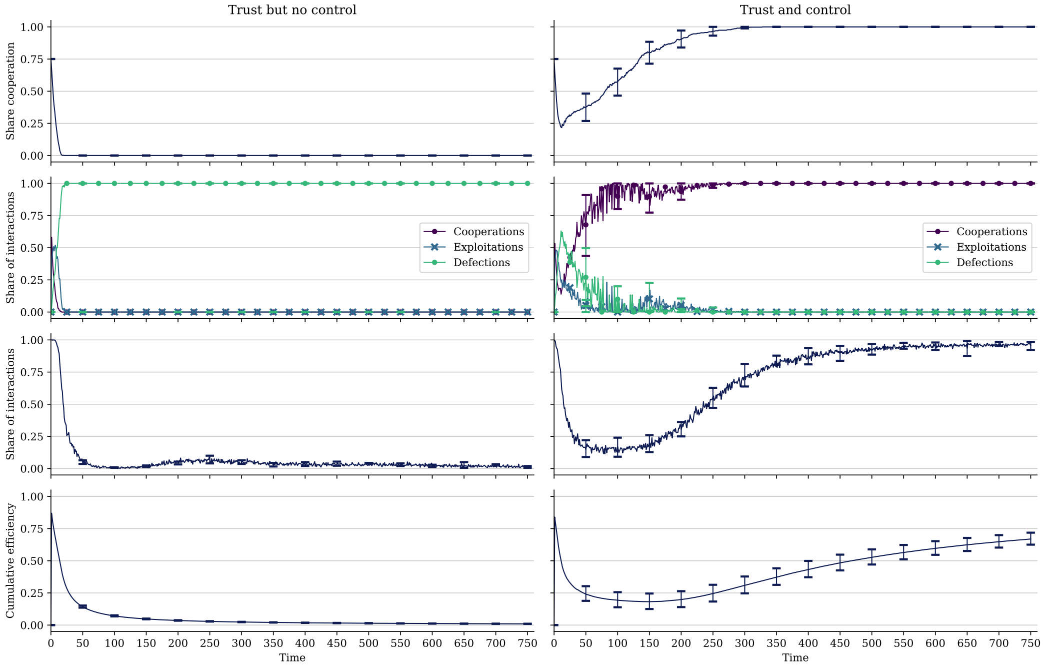


We now turn to the dynamics of the cases in which shocks hit the system. Figure A3 is a slightly extended version of figure 7 in the main text and illustrates the causal effect of the shocks on the model dynamics.

Finally, figure A4 illustrates the similarity between the trust shock and the complete shock. This shows how the trust shock "trumps" the control shock. At the same time, particularly with regard to the efficiency of the system, the complete shock still is more severe than the trust shock alone, as indicated in figure A5.

*Figure A3.*

Dynamics after different shocks.

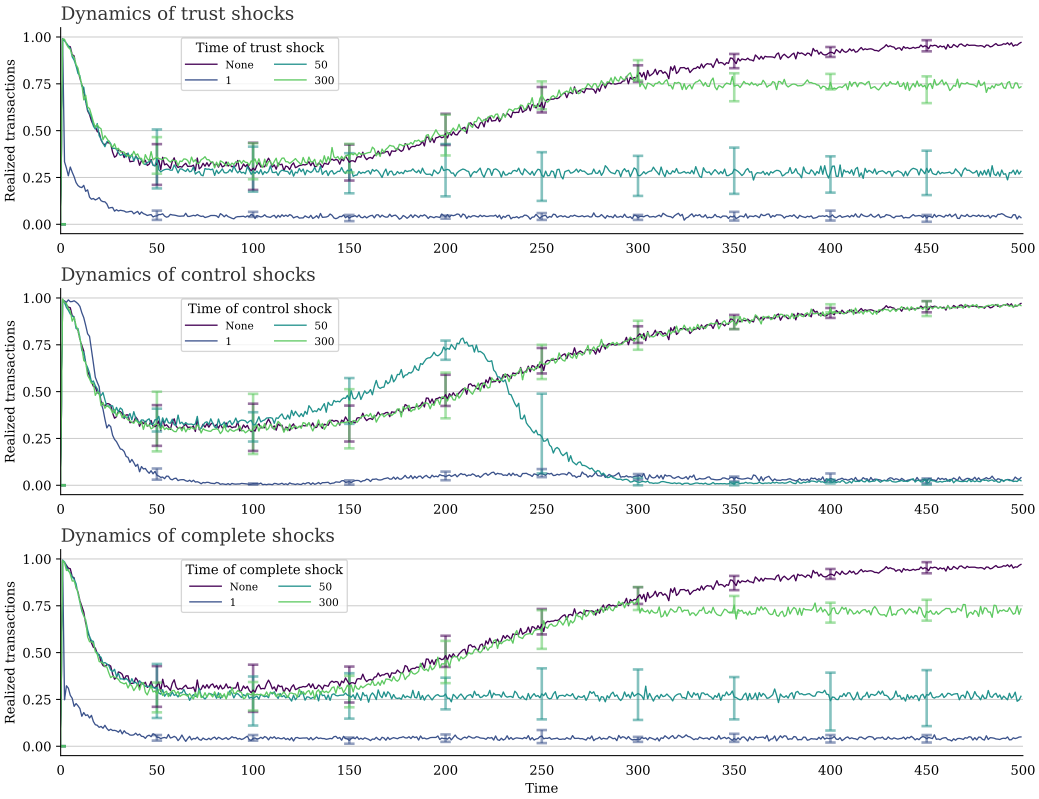


*Figure A4*

Comparing the results of different shocks on the shares of realized transactions. The graph shows the means and 10th and 90th percentiles of 50 simulation runs after 500 time steps with the baseline specification as summarized in table 2 or the main paper.

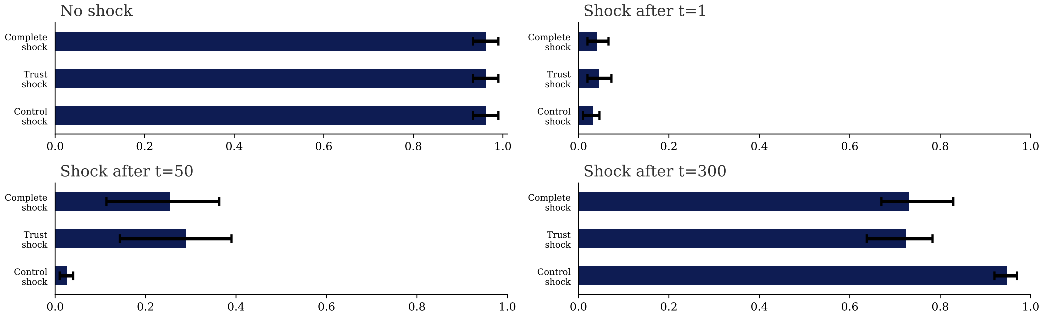

*Figure A5*

Comparing the results of different shocks on the overall efficiency of the system. The graph shows the means and 10th and 90th percentiles of 50 simulation runs after 500 time steps with the baseline specification as summarized in table 2 or the main paper.

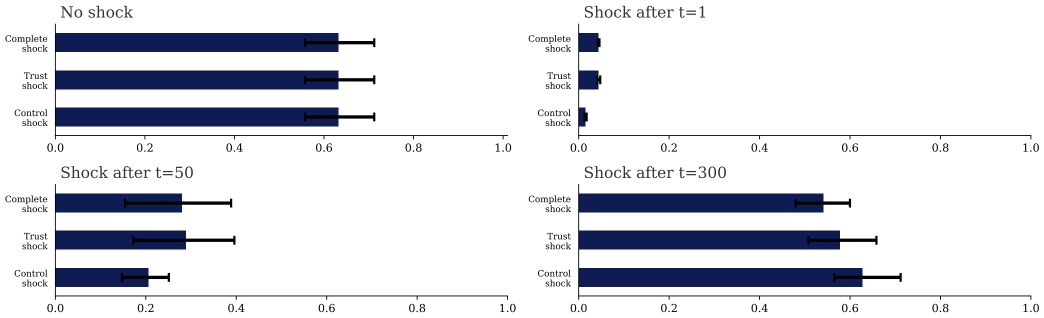


## *S.2 Further sensitivity analysis*

The hawala system is complex and if one wishes to capture all its essential mechanisms in a formal model one has to sacrifice analytical tractability.[1] To partly remedy this drawback, our model has been designed such that the number of free parameters remains as small as possible. We were able to test the effect of every parameter on the model outcome. In effect, while ultimate proofs are not feasible for models as complex as ours, we can say confidently that the model has been verified via extensive statistical analysis of the results. Here we present some further sensitivity analyses of our model.

### *S.2.1 The initial share of cooperators*

With regard to the initial share of cooperators we would expect that too many selfish players prevent the emergence of a functioning hawala system. We suppose that real hawala systems with more than 50% selfish hawaladars would not have a chance of survival because they fail to collect a *minimum critical mass* of cooperative agents (c.f. Elsner and Schwardt 2013). Figure A6 confirms this for our model: while a functioning hawala *may* emerge already with about 40% of honest hawaladars in the beginning, only for shares

[1] A more detailed discussion of verification issues in computational models from a more epistemological perspective can be found in Gräbner (2018).

slightly above 50% such an emergence is guaranteed.[2] Notably, it does not matter too much whether there are 55% or 85% honest hawaladars in the beginning. This illustrates the effectiveness of the self-governing mechanisms explored in our study.

### *S.2.2 The fierceness of the dilemma*

It is an obvious corollary from game theoretic results that the more intricate the dilemma structure, the more difficult it is for cooperation to emerge and for the system to function effectively. In other words: If the relative payoff of betraying the others becomes higher *ceteris paribus*, more agents would be willing to cheat. Because we want to take the perspective of cooperative hawaladars and because we fixed $c = 0$ (for the reasons discussed in section 4.2) and, following the convention, $b = 2a$, we measure the fierceness of the dilemma with the expression $\varphi = {}^{a}/_{|d|}$. The bigger $\varphi$, the less dangerous is cooperative behavior for the agent because the payoff of mutual cooperation compared to the loss of being cheated increases.

*Figure A6*

The role of initial conditions: If there are more than 50% cooperative hawaladars in the beginning, the system almost surely functions well. For less than 50% cooperative hawaladars in the beginning, an efficient system is unlikely. The values on the y-axis are medians and the 10 and 90% percentiles of 10 simulation runs.

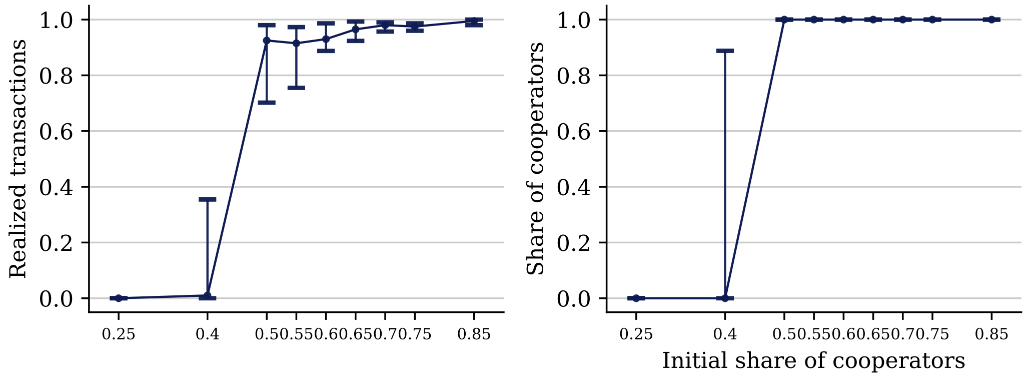


[2] This requirement share could probably be further reduced if a stronger form of preferential attachment was implemented.

The results are presented in figure A7 We observe that our positive results hold for a wide and sensible range of $\varphi$ but that hawala cannot function if the relative cost of getting exploited gets too high. Changing $\varphi$ is an important entry gate for policies that aim at stabilizing systems similar to IVTS, although changing the game form as such is usually not straightforward. Also changing the game form is usually politically costly, and as a theoretical solution to the problem of Hawala rather trivial.

### *S.3 Reasons for the competitiveness of hawala*

Why do people use an informal value transfer system such as Hawala despite the informality and legal unenforceability of financial claims among hawala participants, the obscurity and impenetrability of the system's workings and plentiful opportunities for getting swindled out of one's money, and despite the existence of well-established rivals, like banks, wire transfer companies and smartphone payment systems?

*Figure A7*

The role of the fierceness of the dilemma: If the loss of getting exploited is too high in relation to the payoff from mutual cooperation, hawaladars do not cooperate enough to build up a functioning system. The value of φ is represented on the x-axis. The bigger φ, the less dangerous it is too cooperate.

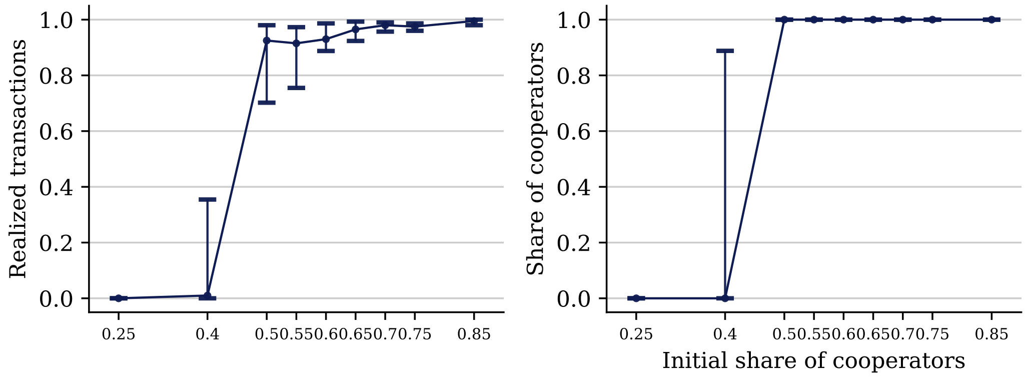


In fact, Hawala can boast significant competitive advantages over its rivals, particularly in those aspects, which are relevant to its main target clientele, migrant workers sending money back to their home countries. Most importantly, Hawala manages remittance flows in distant or dangerous places where the formal banking infrastructure cannot be deployed for security or profitability reasons. Hawala networks offer an equally suitable option for

those customers in the developing countries who lack trust in the official banking services or cannot consume them due to illiteracy, inadequate transportation means or missing paperwork (Razavy 2005). Hawala is less expensive than competing mechanisms of money transfer, with low commission fees and favorable exchange rates being sustained through the lower overhead and compliance costs (Liargovas and Repousis 2011; Qorchi 2002; Shanmugam 2005; Viles 2008; Zagaris, 2007).

This alternative fund transfer system also proves to be fast and reliable, reaching most international destinations within hours rather than days (Qorchi 2002; Zagaris 2007) and avoiding suspicious losses in transit (Shanmugam 2005), as hawaladars prize and strive to maintain their reputation for speed, efficiency and trustworthiness (Nakhasi 2007; Razavy 2005; Viles 2008). Other benefits of Hawala networks include informality (in contrast with mobile or wire transfers, no registration is needed and money can be sent under an assumed name), spatial and temporal flexibility in accommodating customer demand, cultural affinity with hawaladars and a sense of personal interaction between the sender and intermediary (Liargovas and Repousis 2011; Passas 2005; Qorchi 2002; Razavy 2005; Zagaris 2007).

These comparative advantages of Hawala over conventional fund transfer systems far outweigh its potential drawbacks, such as the lack of tangible evidence that a transaction has actually occurred or the necessity to deal with intermediaries who typically operate 'out of nondescript little shops and bazaars' (Shanmugam 2005).

## *Supplementary references*

Elsner, W., Schwardt, H., 2013. Trust and arena size: expectations, institutions, and general trust, and critical population and group sizes. *Journal of Institutional Economics* 10, 107–134.

Gräbner, C., 2018. How to Relate Models to Reality? An Epistemological Framework for the Validation and Verification of Computational Models. *Journal of Artificial Societies and Social Simulation* 21(3), 8. DOI: 10.18564/jasss.3772

Liargovas, P., Repousis, S., 2011. Underground banking or hawalaand Greece-Albania remittance corridor. *Journal of Money Laundering Control* 14, 313–323.

Nakhasi, S.S., 2007. Western Unionizing the Hawala: The Privatization of Hawalas

and Lender Liability. *Northwestern Journal of International Law and Business* 27, 475–496.

Passas, N., 2005. Law enforcement challenges in hawala-related investigations. *Journal of Financial Crime* 12, 112–119.

Qorchi, El, M., 2002. Hawala. *Finanzas Desarollo* 39, 31–33.

Razavy, M., 2005. Hawala: An underground haven for terrorists or social phenomenon? *Crime, Law and Social Change* 44, 277–299.

Shanmugam, B., 2005. Hawala and money laundering: a Malaysian perspective. *Journal of Money Laundering Control* 8, 37–47.

Viles, T., 2008. Hawala, hysteria and hegemony. *Journal of Money Laundering Control* 11, 25–33.

Zagaris, B., 2007. Problems applying traditional anti-money laundering procedures to non-financial transactions, "parallel banking systems" and Islamic financial systems. J*ournal of Money Laundering Control* 10, 157–169.
